# The Effects of Virtual Reality on Enhancement of Self-Compassion and Self-Protection, and Reduction of Self-Criticism: A Systematic Review

**DOI:** 10.3390/ijerph20032592

**Published:** 2023-01-31

**Authors:** Tomáš Žilinský, Júlia Halamová

**Affiliations:** Institute of Applied Psychology, Faculty of Social and Economic Sciences, Comenius University in Bratislava, Mlynské luhy 4, 821 05 Bratislava, Slovakia

**Keywords:** self-compassion, self-criticism, self-protection, systematic review, virtual reality

## Abstract

Background: Virtual reality used for the treatment of mental health disorders is showing promising potential in clinical practice. Increasing self-compassion and self-protections and decreasing self-criticism have been identified as trans-diagnostic mechanisms helping to build a resilient self. The goal of this systematic review was to provide an overview of research studies about virtual reality while exploring its effectiveness in increasing self-compassion and self-protection and decreasing self-criticism. Methods: On 6 December 2022, titles, abstracts, and, where available, keywords were searched in the following databases: PubMed, Scopus, and Web of Science. The inclusion criteria were: empirical study; quantitative methodology; outcomes measured, including self-compassion and/or self-protection, and/or self-criticism; pre/post and/or control group intervention measures of selected outcomes; participants aged 18 and above; application of virtual reality technology as part of the intervention; full study availability; and English language. Exclusion criteria were: ruminations related only to physical pain; self-protection in the context of physical survival; VR used to increase ruminations. Results: Selection criteria were met by 19 studies (two randomized controlled trials, 16 longitudinal studies, and one case study) with an overall number of 672 participants. Results suggest that VR interventions targeting self-criticism, self-compassion, and self-protection might be effective for non-clinical (self-compassion) as well as clinical (self-criticism and self-protection) samples. Discussion: The present systematic review partially supports the effectiveness of VR interventions on self-criticism, self-protection, and self-compassion. To properly answer the question of its effectiveness, more randomized control trials with larger samples from different populations are needed. The results are also limited by the variability of intervention protocols and the amount of exposure to VR. Other: This work was supported by the Vedecká grantová agentúra VEGA under Grant 1/0075/19. This systematic review has not been pre-registered.

## 1. Introduction

Virtual reality (VR), as a facilitator of psychological interventions for the treatment of mental health disorders, is increasingly showing potential for clinical practice [1,2]. This has been particularly the case in the last few years, as it is only since 2016 that high-quality immersive VR technology has become affordable, making it accessible to wider audiences [3].

VR is defined as a technology that enables an advanced interaction between humans and computers and allows for humans to become immersed in a synthetic environment generated by the computer [4]. Although VR is meant to be an interactive and immersive system by design, the various existing forms of VR on the market can be divided into immersive and non-immersive, as well as interactive and non-interactive VR. Non-immersive VR is usually delivered through a computer screen and tends to come in the form of a three-dimensional computer game controlled via a keyboard, joystick, or some other type of interface [5]. Immersive VR, alternatively, can be described as a life-sized environment that evokes in the user the sensation of actually being part of it [6]. Immersion is usually achieved by blocking out sensory stimuli from the physical world and presenting an alternative three-dimensional digital world through a head-mounted display (HMD) or through an immersive room, such as Cave Automatic Virtual Environment (CAVE) [7]. Interactive VR is a system that is essentially sensitive and responsive to the user’s behavior, facilitating an interaction between the user and objects within this virtual world. Conversely, non-interactive VR does not allow for interaction; the user is more-or-less a passive viewer of a digitally generated environment. A 360-degree video would be considered non-interactive VR: although the user can exploit the full view through movements (e.g., head rotation while wearing a HMD), he or she cannot interact with the content of the presented environment.

VR has the capacity to provide a subjective and illusional experience, i.e., to (to some degree) simulate reality and real-world interactions, which indicates broad scientific and clinical implications. Moreover, VR offers tight control over simulation, reduces the inconsistency of interventions, and, most importantly, allows for the design of tailor-made therapeutically valuable situations that are otherwise (nearly) impossible to recreate in real life [6]. So far, research has been conducted on the clinical use of VR in the treatment of mental health disorders, showing promising results for depression (e.g., [8]), anxieties (e.g., [9]), post-traumatic stress disorder (e.g., [10]), eating disorders (e.g., [11]), pain management (e.g., [12]), autism (e.g., [13]), forensic psychiatry (e.g., [14]), and schizophrenia (e.g., [15]).

Depression and anxiety are two of the most common mental health disorders globally [16]. They frequently coexist—depressive people often have symptoms of anxiety, just as people with anxiety disorders often suffer from depression [17]. One of the main risk factors predicting both depression and anxiety has been identified as self-criticism [18]. Self-criticism can be conceptualized as an intense and persistent relationship one has with oneself, consisting of an unshakeable and increasing demand to constantly perform within the highest standards and an inwardly-directed hostility and depreciation when this unachievable demand is not met [19]. Self-criticism is often interchangeably used with or related to negative ruminations, self-judgement, self-hatred, self-contempt, self-attack, the inner critic, or self-shame. As the antidote to the pain that self-criticism (or negative self-treatment) causes, two adaptive processes were identified—self-compassion and self-protection—both within the framework of Emotion-focused Therapy [20]. In the therapeutic model of Emotion-focused Therapy [21], self-criticism, self-compassion, and self-protection are meaningfully tied together, and working with them is central to building one’s empowered and resilient self [20]. Emotion-focused Therapy’s efficiency for the treatment of self-criticism was supported, for example, by Shahar et al. [22,23], Thompson and Girz [24], and Timulak et al. [25].

Self-compassion is best understood as inwardly-directed compassion [26]. It can be conceptualized as an emotion [27,28], frame of mind [29], skills set [30], or process [31] that is associated with understanding the universality of human suffering, recognizing one’s own suffering, feeling for the suffering person, tolerating uncomfortable feelings, and acting on or having the motivation to act on that suffering in order to alleviate it. Interchangeably with self-compassion, scholars also use terms such as self-soothing or self-reassurance. Self-protection can be understood as assertiveness directed at one’s own inner critic. Also labelled as assertive anger [32] or protective anger [20], self-protection can be defined as a state or adaptive emotion that allows a person to stand up for their rights and needs, set boundaries against, and assertively engage in a fight with their own mistreatment they have internalized from the voices of important figures, mainly in childhood. More recently, Neff [33] introduced a similar concept of fierce self-compassion, which is defined as a united caring force against environmental pressures and injustices but also against one’s own habits. Interest in self-compassion has been growing significantly since the early 2000s [34], with the first few studies employing VR to enhance self-compassion appearing more recently (e.g., [35]). So far, it seems that a lot less has been published about self-protection in general. However, assertiveness most likely entered VR research at the turn of the millennium [11].

This systematic review focuses on VR interventions targeting the three abovementioned self-concepts: self-criticism, self-compassion, and self-protection. This systematic review summarizes the existing scientific literature on VR interventions applied to decrease self-criticism or increase self-compassion or self-protection, compared with control treatment, no treatment, or baseline scores in the clinical and non-clinical adult populations. The results of this systematic review may inform the development of effective VR interventions targeting not only these three self-concepts but ultimately may lead to new ways of addressing depression and anxiety. To our knowledge, this is the first systematic review focusing on self-compassion, self-criticism, and self-protection applied in virtual reality. As results of using virtual reality for mental health interventions are very promising, and these three concepts play a pivotal role in psychopathology as well as mental and physical health, we were particularly interested in how effective VR is for these particular three concepts.

## 2. Methods

This systematic review was directed according to the Preferred Reporting Items for Systematic Review and Meta-Analyses (PRISMA), using the 2020 updated guidelines [36].

### 2.1. Information Sources

This systematic review includes all literature written in English, published until 6th December 2022, and accessible through the PubMed, Scopus, and Web of Science databases. The main focus was on VR as a platform facilitating the increase of self-compassion and/or self-protection and/or the decrease of self-criticism. In all three databases, records (titles and abstracts) were searched, and where available, keywords were added to the search too.

### 2.2. Search Strategy

The searches were completed in three separate steps, depending on the search topic. The general search terms for all three searches were: (“virtual reality” OR “VR” OR “virtual environment*” OR “virtual world*” OR “avatar” OR “serious game*”). The topic-specific search terms for self-compassion were: (“compassion*” OR “self-compassion*” OR “self-sooth*” OR “self-reassur*”); for self-criticism: (“self-critic*” OR “inner critic*” OR “self-contempt*” OR “ “self-hat*” OR “self-attack*” OR “ruminat*” OR “self-judg*”); and for self-protection: (“self-protecti*” OR “protective anger” OR “assertive anger” OR “assertive*” OR “self-assert*” OR “fierce self-compassion”). See Appendix A for the full search syntax used in each of the databases.

### 2.3. Eligibility Criteria

The inclusion criteria were: empirical study; quantitative methodology; outcomes measured, including self-compassion and/or self-protection/assertiveness, and/or self-criticism/negative ruminations; pre/post and/or control group intervention measures of selected outcomes; participants aged 18 and above; application of VR technology as part of the intervention; full study availability; and English language. Exclusion criteria were: ruminations related only to physical pain; self-protection in the context of physical survival; and VR used to increase ruminations. When synthesizing the evidence, studies were grouped according to the main outcomes of this review: self-criticism, self-compassion, and self-protection.

### 2.4. Selection Process

The eligibility criteria were decided on by both authors. The database search was conducted by the first author (TŽ). The screening was completed by the first author under full supervision of the second author (JH). The first author justified all selected and rejected studies to the second author. The second author challenged the first author’s justifications. All decisions on inclusion and exclusion were made consensually by both authors.

### 2.5. Data Collection

All variables were agreed on by both authors. The data collection process was conducted by the first author and checked by the second author. The first author consulted the second author about any uncertainties or inconsistencies that arose from the data collection process. Only variables relevant to this systematic review were extracted. If other measurements had taken place in the selected studies, these were not included. In total, nine variables had been decided: one outcome domain (#8) and 8 additional variables (#1-#7, #9):

1. Type of the study design; studies were defined according to the purpose of this systematic review (i.e., even though a study had a control group, if the control group was not used as a comparison for the intervention group in respect to our outcomes, we treated it as a within-group study).
2. The components of each intervention and, where available, the duration of each component; and the total duration of VR exposure one participant in the intervention group received.
3. Whether participants in the control group received VR exposure too or not.
4. The content of each VR intervention.
5. Immersion and interactivity of VR technology used.
6. The type of sample used in the study and their age.
7. The size of intervention and control group(s).
8. What outcomes, according to the focus of this systematic review, were measured (self-compassion, self-protection, assertiveness, negative ruminations, or self-criticism) and how (names of relevant measuring tools used, if available).
9. The results of relevant outcomes as measured in point #8.

Not all studies reported all variables in full detail. Where available, study investigators were contacted directly via email for any missing information. We define only immersive/non-immersive and interactive/non-interactive VR technology as, likely, this is the most straightforward way to understand VR technology from a non-technical user experience point of view. There are, of course, other parameters (e.g., display resolution, manufacturer, etc.) that could be looked at when classifying VR technology; however, this would perhaps make a lot more sense once there are more studies published.

### 2.6. Risk of Bias and Certainty Assessment

The risk of bias was assessed by the first author and checked and challenged by the second author. Discrepancies were discussed, clarified, and agreed upon together. As a framework, we followed the Cochrane Collaboration’s tool for assessing risk of bias in interventions [37], looking at selection, performance, detection, attrition, reporting, and other biases. We used this tool for both randomized and non-randomized studies. To assess attrition, we followed the five-and-20 rule of thumb used by some researchers, considering attrition of less than 5% as posing little threat and attrition greater than 20% as posing a potentially serious threat to the results’ validity [38].

Additionally, the certainty assessment for all outcomes was assessed by the first author and checked and challenged by the second author. Discrepancies were discussed, clarified, and agreed upon together. Certainty assessment was completed using the Grading of Recommendations Assessment, Development, and Evaluation (GRADE) framework [39], used for rating the quality of evidence in systematic reviews. The risk of bias due to missing results (publication bias) was assessed as part of the GRADE framework [39].

## 3. Results

A total of 468 records were identified through our database searches. The exact search breakdown can be seen in Table 1. Selection criteria were met by 19 articles, of which two articles related to seemingly identical research [40,41]; hence, the final selection list is composed of 18 articles. However, one study [42] included different measurements of relevant variables at different time points: one measurement with one particular psychometric tool (MSCS; [43]) was taken at time points T1 and T4, and a different measurement using a different psychometric tool (SOFI; [44]) was taken at time points T2 and T3. In this case, we would treat them as two separate studies, making the final total of selected studies 19. See Table 2 for the complete list of studies in alphabetical order and the structured summaries for each variable.

**Figure 1:**
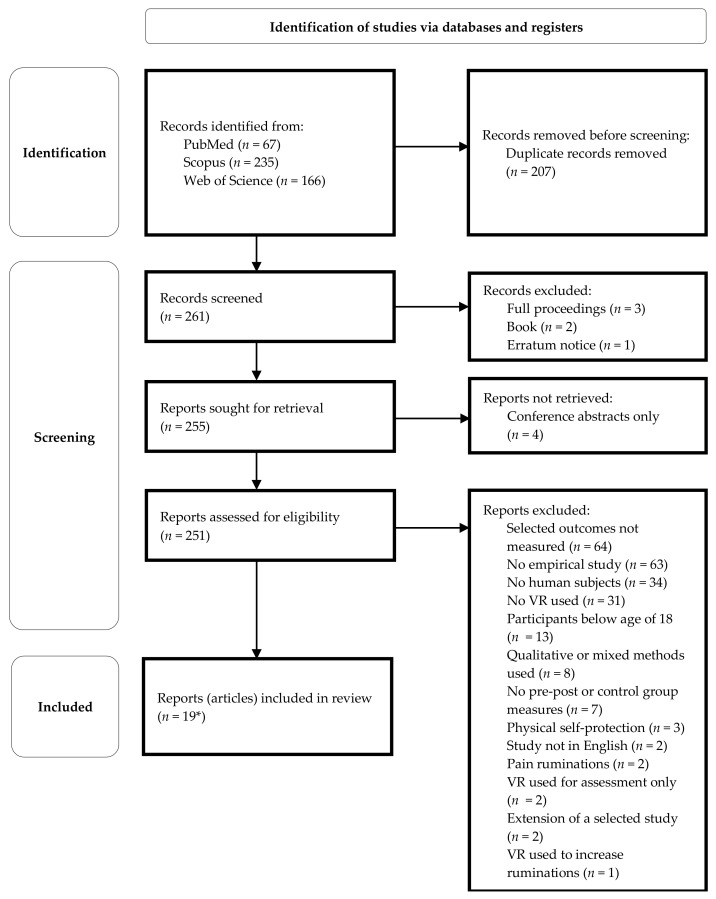
PRISMA 2020 flow diagram of selected reports. * Two articles on the final list had been identified as reports of the same research; therefore, the final number of selected reports is 18.

The most common reasons for reports not to be selected for this review were: selected variables not measured; no empirical study; no human subjects involved; VR not used; participants below the age of 18; and no pre/post and/or control group intervention measures of selected outcomes. For details, see the PRISMA flow diagram in Figure 1.

In terms of study designs, we looked at the studies from the perspective of our outcomes (self-compassion, self-protection, and self-criticism). If a study had a control group, but this control group was irrelevant to the outcome of our interest, we would treat it as a within-group study rather than a between-group study. Of the 19 selected studies, two were randomized controlled trials, 16 were longitudinal studies, and one was a case study. Of the 16 longitudinal studies, seven were randomized between-group studies, two were non-randomized between-group studies, one was a between-group study with randomization not specified, and six were within-group studies (see Table 2 for details). 

Overall, in terms of bias, each of the included relevant studies shows between none and two low risks of bias, three to five high risks of bias, and one to three unclear risks of bias out of the six predefined categories of bias [37]. For self-criticism, three to five high risks of bias and one to three unclear risks of bias were identified amongst the respective studies. For the self-compassion domain, three to five high risks of bias and one to three unclear risks of bias were identified among the respective studies. Lastly, for the self-protection domain, three to four high risks of bias and one to two unclear risks of bias were identified amongst the respective studies. This indicates that some concern is certainly needed when interpreting results across each of the domains of this review (see Appendix B for details and Table 3 for a summary). In terms of certainty assessment, all three domains were rated as very low, mainly due to the dominance of small studies that have been conducted to date and the large variability amongst them (see Table 4 for details).

Across all the studies, the overall number of participants taking part was 672, with 351 in the intervention and 321 in the control groups (these counts are only estimations as two studies did not provide the exact ratio between the intervention and the control group; [11,55]). Since some participants took part in two studies [42], we included them twice in our total count. Three studies [47,48,53] had a control group that was not relevant for measuring our outcomes; these participants were not included in the abovementioned counts. If a study had more than two groups, the group that received VR intervention was considered the intervention group, and the remaining groups were considered the control group. See Table 2 for details.

The final synthesis was grouped around each of the outcome domains: self-criticism, self-compassion, and self-protection. If a study measured two outcomes, e.g., self-criticism and self-compassion, the respective variables (as shown in Table 2) were synthesized for each outcome separately.

### 3.1. Self-Criticism

This systematic review identified six studies within the self-criticism outcome domain, with three of them measuring the impact of VR on self-criticism [8,35,50] and three on ruminations [47,48,54]. Of the six studies, four were published from 2016 onwards. Chronologically, the first study for this outcome looked at the application of VR to post-event processing in people with social anxiety disorder [54]. All six studies showed a decrease in self-criticism post-intervention; in five cases [8,35,47,48,54], this decrease was statistically significant. Additionally, four studies reported large effect sizes for self-criticism and ruminations decrease [8,35,47,50]; one study reported a large effect for ruminations decrease as part of a larger treatment group [54]; and one study did not report an effect size for ruminations at all [48].

All six studies used immersive head-mounted displays and interactive scenarios, and VR comprised the central part of all interventions. Four studies [8,35,47,48] used psychoeducation as part of the intervention, and three studies [8,35,50] applied embodied experience. On average, they had 24 (between 15 and 32) participants in the intervention groups. Four studies used clinical populations; in the remaining two cases [35,50], the populations were non-clinical. Three studies [8,47,48] did not have a control group, and one study [35] applied VR in the control group as well. The data on the number of completed VR sessions and overall durations spent in VR is not complete; from those reported, participants received between one and eight VR sessions with an overall VR exposure of 9.5 to 90 min. The scenarios involved either comforting a virtual child, embodying a patient with a severe anxiety disorder, or introducing oneself to or speaking to a virtual audience varying in size and level of disruption.

Results suggest that using immersive and interactive VR technology could be effective in targeting self-criticism. Additionally, psychoeducation may possibly play its part too as it was part of the intervention in four out of six studies; however, there is not enough evidence to either support or refute this observation. All studies with clinical samples showed significant results. All studies targeting the ruminations of participants suffering from social anxiety disorder (through virtual anxiety-provoking situations) reported significant reductions in ruminations. Where reported, studies with significant results delivered a minimum of three VR sessions with an overall VR exposure per participant of 24 min.

### 3.2. Self-Compassion

In terms of the outcome of self-compassion, nine studies were identified; eight of them measured self-compassion [8,35,42,45,49,50,51,53]; and one measured positive qualities towards self as a measure of self-compassion [42]. All studies, except for one [35], were published from 2016 onwards. Chronologically, the first study in this outcome domain applied VR embodiment experience to decrease self-criticism through self-compassion in women high in self-criticism [35]. Overall, of the nine studies, seven showed an increase in self-compassion [8,35,42,45,49,53]; for five of them, this increase was significant [8,35,45,49,53]. Of the remaining two studies, one showed a non-significant decrease in self-compassion [50] and one showed a non-significant change in self-compassion without reporting the direction of the effect [51]. Five studies [8,35,42,49] reported large effect sizes for self-compassion; one study [50] reported no effect for self-compassion; and three studies [45,51,53] did not report any effect size.

Of the nine studies in this domain, six [8,35,42,45,50] used immersive and interactive technology; two [49,51] used immersive head-mounted displays with non-interactive scenarios; and one [53] employed a non-immersive laptop with interactive content. In two studies, however, VR did not constitute a central part of the intervention [42,53]; in fact, it could be said that VR was marginal compared with the much larger proportion of other intervention parts. Embodied experience was part of the VR intervention in six studies [8,35,42,45,50]. Participants attended between one and six sessions, with all but three studies comprising only one VR session. Where reported, participants engaged in a minimum of 4 to 46 min of VR experience. The samples were, except for two studies [8,51], non-clinical, with an average intervention group size of 19 (between eight and 70). Three studies [8,51,53] had no control group, and two studies [35,45] had their control group also exposed to VR. Scenarios varied from an astronaut’s mission to the moon, observing people, avatars, objects, or nature, to embodying a child or an adult, healthy or with a mental condition.

Results suggest that VR could be effective in increasing self-compassion; looking at studies with significant results, this could be true even after just one 10 min, potentially even shorter, VR session. However, as all of the samples except for two were non-clinical, this can be concluded only for non-clinical adult samples. Technology, scenarios, and the structure of interventions varied across the studies.

### 3.3. Self-Protection

For this outcome domain, seven studies were identified; all of them measured the impact of VR on assertiveness [11,15,40,41,52,55,56,57]. All the studies were published before 2016. Chronologically, the first study in this outcome domain applied VR to treat body image disturbances in overweight women [11]. All seven studies showed a positive impact of VR on assertiveness; five studies [11,15,40,41,52,55] showed significant improvements; one study [57] fully reported only one measure, and it showed significant improvement for assertive behavior; and one study [56] did not report any inferential statistics. In terms of effect sizes, one study [57] reported a large effect for one of their assertiveness measures but did not report an effect for their second assertiveness measure; one study [40,41] reported a small and medium interaction effect between VR and CBT groups for their two assertiveness measures; another study [52] reported a large effect of time and a medium interaction effect between their VR and non-VR training groups; the remaining four studies did not report any effect sizes.

All seven studies employed interactive scenarios; however, only three of them [11,52,55] used head-mounted displays; the rest used personal computers, although in two cases [15,57] together with 3D glasses and headphones. In all studies, VR was a central part of the intervention. All participants attended between seven and 16 sessions, with each receiving approximately 220 to 480 min of VR experience in total; in one study [52] this information was not provided. VR was used only in intervention groups, which consisted solely of clinical samples averaging 13 participants (between one and 32). All studies but one [15] had a control group, and one study [57] was a case study of one patient. Scenarios included daily social situations and exposure to challenging stimuli.

Results suggest that VR could be effective in increasing self-protection/assertiveness, specifically when using VR technology with interactive scenarios that elicit challenging emotions in different virtual situations. However, based on this systematic review, this can be concluded only for clinical populations. Where reported, studies with significant results show a minimum of seven VR sessions with an overall VR exposure per participant of 220 min.

### 3.4. Adverse Effects

In addition to the analysis above, we also screened the studies in this review for any reports of adverse side effects as a result of taking part in VR, also known as cybersickness [70]. One study [51] reported adverse side effects (nausea and dizziness) in two participants; however, not necessarily attributable to VR intervention. Three studies [11,52,55] reported no cybersickness in any of their participants. The remaining studies did not address VR-related adverse side effects in their studies.

## 4. Discussion

This systematic review looked at the effectiveness of VR in decreasing self-criticism and increasing self-compassion and self-protection. Positive results have been identified across all three domains, adding to the encouragement of the application of VR for mental health purposes [5,6]. However, there are apparent differences (study design, number and duration of VR exposure, technology, scenarios, etc.) in the reviewed studies, and careful considerations need to be taken into account when interpreting these findings and offering any conclusions.

For decreasing self-criticism, immersive VR technology with interactive scenarios shows positive results after a relatively short-term intervention, specifically for adult clinical samples. This, or any other broader judgment, is however very limited, considering there were only six studies identified for this domain. It appears that this area has so far not received enough attention, considering the first study appeared in 2011. In addition, if there is a significant impact on clinical samples, it would be worth testing efficiency in non-clinical samples as well because the impact could be potentially even higher, as clinical samples may struggle more to overcome self-critical thoughts [71].

A brief VR intervention could be effective for increasing self-compassion in adult non-clinical samples. This conclusion comes from reviewing nine studies. These studies, however, varied greatly, indicating that more research is needed to better understand which features of VR intervention make it effective. An area to closely look at might be embodied experience, as already pointed out by Falconer et al. [35]. It seems promising that some of the studies with only one VR exposure made significant changes to self-compassion. If further elaborated, there is a big potential for improving the well-being of a greater non-clinical population. In addition, we suggest testing its potential with clinical samples as well.

For increasing self-protection/assertiveness, VR with interactive scenarios seems to have a positive effect, specifically in adult clinical samples. This, or any other conclusion, is again very limited, considering there were only seven studies identified for this domain. What stands out for this domain is that more repetitions and longer overall VR exposure took place compared with the other two domains. However, it is unclear whether fewer repetitions and a shorter overall exposure would have a different effect. With only seven studies to date, this area of research seems to have received very little attention since its onset in 2001, with the latest study to date published in 2014. It would be worth testing the impact of just one, or a shorter, VR exposure to develop more cost-effective interactions for increasing self-protection.

Interventions varied greatly across all the studies, and it remains unclear what is/are the key component(s) of effective VR interventions. Looking at the technology employed, the majority of the studies used immersive HMD, with only a few using a non-immersive computer screen. As none of the non-immersive studies provide information on the size of the effect, it is hard to judge what impact computer immersion plays on intervention effectiveness. Similarly, little can be concluded about interactivity, as only two studies provided non-interactive, passive content; the vast majority of the studies had their participants actively involved in the process of intervention. Additionally, the actual VR scenarios varied considerably in terms of content, number of exposures (repetition), and time spent in VR. This follows the observation of Rizzo and Koenig [5] that the identification of active ingredients in the use of clinical VR is still very much needed.

Another area of concern is the sample size. Small sample sizes can make statistical significance and effect size results unreliable. Field et al. [72] suggest a sample size of approximately 30 as a threshold for a reliable real-world population estimate. In our review, in all three domains, the average sample size was below this number, making any generalizations difficult. Additionally, more than a third of the studies were conducted on students, further adding to this issue. Larger sample studies with various population types are needed.

Furthermore, a great variety of self-report measures have been used across the selected studies, making a reliable synthesis difficult. Additionally, many of the selected studies show an overreliance on self-reported measures. Self-rated scales inevitably produce errors [72] and are confounded by bias, as reported by some of the authors in this review too (e.g., [48]). Therefore, the interpretation of results needs to take this point into account. Wider employment of other means of measurement (e.g., behavioral, vocal, or physiological markers) could prove beneficial for future research in this area. Additionally, reporting adverse effects, even with zero occurrence, should become more common for VR research, as it is unclear whether they appear only very rarely or they simply go unreported.

The results of this review predominantly focus on short-term change, with longer-term effects unknown. With only three studies employing follow-up measurements, the retention of positive changes beyond immediately after the intervention needs further research in order to establish the effectiveness of VR for our domains.

Since self-compassion and self-protection were identified as the antidotes to self-criticism within the latest research findings of Emotion-focused Therapy [20], we propose developing a novel VR intervention to decrease levels of self-criticism through evoking self-compassion and self-protection based on the two-chair technique [73,74].

Consistent with the wider affordability of VR as of 2016 [3], the number of studies looking at self-compassion and self-criticism has significantly grown this year. However, this is not the case for self-protection/assertiveness, as the latest study in this domain was published in 2014. This highlights a large research gap and an imminent need to expand on this area, especially if published studies suggest an existing potential. In fact, considering the low overall number of studies in this review, this statement can be applied to all three domains. More studies, especially randomized controlled trials (there were only two in this review), are needed to investigate the effect of VR on self-criticism, self-compassion and self-protection. Future trials should most importantly include larger samples, both clinical and non-clinical, and look at longer-term effects through follow-up periods; additionally, VR interventions need to be replicable and clearly embedded in theory to stimulate wider research; and finally, since age (e.g., [75]) and gender (e.g., [76]) may play a part in the way VR experience is processed, these should be investigated too.

## 5. Limitations

This systematic review has been conducted by only two reviewers. There is always a possibility that some studies may have been missed during the process. Limited published literature limits broader conclusions. It is difficult to estimate how many studies containing non-significant results may have gone unpublished or were published in different languages except for English. Not all studies reported all details, making a sound judgment difficult, and not all of the contacted authors responded to our questions to specify their research study. The selected studies varied greatly, leaving the final synthesis open to doubt. Finally, this study was not pre-registered. For future systematic reviews looking at this research area, we suggest more reviewers be involved and pre-register the protocol in order to mitigate any additional bias that may have arisen from these limitations.

## 6. Conclusions

A systematic review, guided by the PRISMA 2020 principles [36], has been conducted in order to provide an overview of the effectiveness of VR interventions aiming to increase self-compassion and self-protection and decrease self-criticism. In all three domains, positive results have been identified, supporting the notion that VR will become more established in mental health practice in the near future. However, any generalizations have to be inferred very carefully because the number of studies identified in this review is limited and they vary in design and application. Larger sample randomized controlled trials with a longer term follow-up period are required in order to establish robust evidence for VR interventions for all three outcome measures.

## Figures and Tables

**Table 1:** Breakdown of search records according to topic and databases applied.

	Virtual Reality + Self-Compassion	Virtual Reality + Self-Criticism	Virtual Reality + Self-Protection	Σ
PubMed	28	16	23	67
Scopus	90	46	99	235
Web of Science	79	34	53	166
Σ	197	96	175	468

All data is shown before duplicates were removed.

**Table 2:** Final summary of selected studies.

Reference	Study Type (Defined According to the Purpose of This Systematic Review)	Intervention (Length in Minutes)/Overall VR Exposure in Minutes	VR Exposure in Control Group(s)	VR Intervention Description	VR Type	Sample (age)	Intervention/Control Groups Size	Selected Outcomes (Measure)	Results of Outcomes
Ascone et al., 2020 [45]	Randomized, longitudinal (between-groups) study	Psycho-education + VR exposure with embodiment (10)/10	Yes	Mission to the moon to explore mysterious interactive nebula	Immersive (HMD)/interactive	Students with mildly elevated paranoia symptoms (18+)	12/9	Self-compassion (brief, state-adapted SCS based on [46])	Intervention and control groups analyzed independently (within-group). Significant positive change in self-compassion post-psycho-education and VR exposure in intervention group (*χ^2^* = 16.93, *p* < 0.001). No significant change in self-compassion in control group (*χ^2^* = 3.16, *p* = 0.206). No effect size reported.
Cebolla et al., 2019 [42] (7A) (T2,T3)	Randomized, longitudinal (between-groups) study	Virtual body swap/embodiment I. (5) + Self-compassion audio meditation (15) + Virtual body swap/embodiment II. (5–7)/10–12	No	I. Being in someone else’s body; II. Hugging oneself from third-person perspective	Immersive (HMD)/interactive	University students (18+)	8/8	Compassion toward self (positive qualities towards self) (SOFI)	Increase in positive qualities towards self; however, no significant interaction between intervention and control groups, [*F*(1, 14) = 0.66, *p* = 0.429, *η^2^p* = 0.05]. Large within-group effect size for positive qualities towards self in intervention group (*d* [95% CI] = −0.82); CI for control group included 0.
Cebolla et al., 2019 [42] (7B) (T1,T4)	Randomized, longitudinal (between-groups) study	Virtual body swap/embodiment (5) + Self-compassion audio meditation (15) + Virtual body swap/embodiment (5–7) + 2 weeks of voluntary self-compassion audio meditation/10–12	No	I. Being in someone else’s body; II. Hugging oneself from third-person perspective	Immersive (HMD)/interactive	University students (18+)	8/8	Self-compassion as part of self-care behaviors (MSCS)	Increase in self-compassion with no significant interaction effects between intervention and control groups for Self-compassion and Purpose subscale [*F*(1, 14) = 1.34, *p* = 0.266, *η^2^p* = 0.09]. Large within-group effect size for this subscale in the intervention group (*d* [95% CI] = −0.77); CI for control group included 0.
Falconer et al., 2014 [35]	Longitudinal (between-groups) study; information on group randomization not reported	Psycho-education + Embodiment as an adult (3) + VR scenario I. + Embodiment as a child (3) + VR scenario II./not all durations reported	Yes	I. Speaking to a distressed virtual child; II. Receiving one’s own soothing message from the child’s perspective	Immersive (HMD)/interactive	Female undergraduate students with higher levels of self-criticism (18+)	22/21	State self-compassion (SCCS), state self-criticism (SCCS)	Self-compassion increased in 1PP group; significant main effect of time [*F*(1, 41) = 14.33, *p* < 0.001, *η^2^p* = 0.26], with significant interaction between 1PP and 3PP groups [*F*(1, 41) = 15.87, *p* < 0.001, *η^2^p* = 0.28]. Self-criticism decreased in both 1PP and 3PP groups; main effect of time [*F*(1, 41) = 42.1, *p* < 0.001, *η^2^p* = 0.51], with no significant interaction between 1PP and 3PP groups [*F*(1, 41) = 3.46, *p* = 0.07, *η^2^p* = 0.8].
Falconer et al., 2016 [8]	Longitudinal (within-group) study with 4-week follow-up	Psycho-education + 3× once weekly [Embodiment as an adult (2) + VR scenario I. (2) + Embodiment as a child (2) + VR scenario II. (2)]/24	N/A	I. Speaking to a distressed virtual child; II. Receiving one’s own soothing message from the child’s perspective	Immersive (HMD)/interactive	Current major depressive disorder (18+)	15/-	Self-compassion, self-criticism (SCCS)	Significant linear increase in self-compassion [*F*(1, 12) = 6.65, *p* < 0.02, *η^2^p* = 0.36] and significant linear decrease in self-criticism [*F*(1, 12) = 23.41, *p* < 0.001, *η^2^p* = 0.66] between pre, post, and 4-week follow-up.
Hur et al., 2021 [47]	Longitudinal (within-group) study	6× [Introduction & VR meditation (5) + VR exposure (7–8) + VR meditation & psycho-education (3)]/90	No	Participants introduce themselves in student group meeting. Scenarios vary in difficulty.	Immersive (HMD)/interactive	Social anxiety disorder (18+)	21/21 (control group not relevant for selected variable)	Negative post-event ruminations (PERS)	Negative post-event ruminations significantly decreased between pre- and post-measures (*Z* = −3.32, *p* < 0.001, *r* = 0.51).
Kim et al., 2020 [48]	Longitudinal (within-group) study	6× [Introduction & VR meditation + VR exposure + psycho-education]/90	No	Participants introduce themselves in student group meeting. Scenarios vary in difficulty.	Immersive (HMD)/interactive	Social anxiety disorder (18+)	32/33 (control group not relevant for selected variable)	Negative post-event ruminations (PERS)	Negative post-event ruminations significantly decreased after the intervention [*F*(2.730) = 6.97, *p* < 0.001]. Effect size not reported.
Klinger et al., 2004; 2005 [41,40]	Non-randomized, longitudinal (between-groups) study	Introduction + 11× [VR assessment and/or VR exposure (20)]/220	No	Four different social situations causing anxiety.	Non-immersive (PC)/interactive	Outpatients with social phobia (18+)	18/18	Assertiveness (RAS, SCIA)	Assertiveness significantly increased in both VR and group CBT conditions; *F*(1, 34) = 36.30, *p* < 0.001 for RAS and *F*(1, 34) = 65.77, *p* < 0.001 for SCIA/Assertiveness subscale; CBT shows greater improvement for both subscales. No significant interaction effects were found; *F*(1, 34) = 2.66, *p* > 0.05, *η^2^* = 0.07 for RAS, and *F*(1, 34) = 0.81, *p* > 0.05, *η^2^* = 0.02 for SCIA/Assertiveness subscale.
Modrego-Alarcón et al., 2021 [49]	Randomized controlled trial with 6-month follow-up	6× once weekly [Mindfulness meditation (75) + VR meditation (app.7.5)]/46	No	Observing objects (tree, leaves, lemon, etc.), human figure, walking through a landscape, taking part in a university exam.	Immersive (HMD)/non-interactive	University students (18+)	70/65/53	Self-compassion (SCS)	Self-compassion significantly increased from pre-to-post (*B* = 7.93, *p* < 0.001, *d* = 0.94) and from pre- to 6-month follow-up (*B* = 12.42, *p* < 0.001, *d* = 1.47) in VR mindfulness group, compared with relaxation. No significant interaction effect between VR and non-VR mindfulness group; pre-to-post (*B* = 0.86, *p* = 0.71, *d* = 0.1) and pre- to 6-month follow-up (*B* = −3.11, *p* = 0.19, *d* = −0.36).
Navarrete et al., 2021 [50] (T2,T3)	Randomized, longitudinal (between-groups) study	Embodiment (5) + Embodiment with audio (4.5) + Audio (3)/9.5	No	Embodying a patient with panic attack disorder and directing compassion towards him.	Immersive (HMD)/interactive	Healthcare students and professionals (18+)	21/20	State self-compassion (VAS-SC, 2 questions), state self-criticism (VAS-SC, one question)	Self-compassion decreased a little in intervention group [*t*(20) = 0.21, *p* = 0.838, *η^2^* = 0.00], no significant interaction with control group [*F*(1, 38) = 0.35, *p* = 0.556, *η^2^* = 0.00]. Self-criticism decreased in intervention group [*t*(19) = 1.85, *p* = 0.079, *η^2^* = 0.15], no significant interaction with control group [*F*(1, 37) = 0.03, *p* = 0.868, *η^2^* = 0.00].
O’Gara et al., 2022 [51]	Longitudinal (within-group) study	3× [VR exposure (10) at least a week apart]/30	N/A	360° video of a beach, animated mountain, or animated forest scene, with choice of a male or female guiding voice delivering breathing and CMT exercises	Immersive (HMD)/non-interactive	Cancer patients (18+)	10/-	Self-compassion (SCS)	No significant changes between baseline, VR1, VR2, and VR3 sessions for any SCS subscales (SK: *χ*^2^(3) = 0.733, *p* = 0.866; SJ: *χ*^2^(3) = 2.133, *p* = 0.545; CH: *χ*^2^(3) = 0.976, *p* = 0.807; IS: *χ*^2^(3) = 2.018, *p* = 0.569; MF: *χ*^2^(3) = 5.23, *p* = 0.156; OI: *χ*^2^(3) = 4.417, *p* = 0.22). No significant changes between baseline and VR3 sessions for any SCS subscale (SK: *Z* = −1.011, *p* = 0.312; SJ: *Z* = −0.978, *p* = 0.328; CH: *Z* = −0.224, *p* = 0.823; IS: *Z* = −1.261, *p* = 0.207; MF: *Z* = −1.605, *p* = 0.108; OI: *Z* = −1.43, *p* = 0.153). Total SCS score and effect size not reported.
Park et al., 2011 [52]	Randomized controlled trial	10× [3× VR role plays, including modeling by therapist and positive or corrective feedback (90)]/duration details not reported	No	Conversation, assertiveness, and emotional expression skills trained in common social situations.	Immersive (HMD)/interactive	Inpatients with schizophrenia (18+)	32/31	Assertiveness (RAS)	Improvements in assertiveness in both VR and non-VR social skills training group; significant time effect, *F*(1, 62) = 26.17, *p* < 0.001, *η^2^p* = 0.3; and significant time x group interaction, *F*(1, 62) = 4.96, *p* = 0.03, *η^2^p* = 0.07. VR group shows greater improvement on RAS score.
Park & Ogle, 2021 [53]	Longitudinal (within-groups) study	4× [Body positivity program (120)] + Virtual avatar experience (4–13)/4–13	No	Presenting anthropometrically accurate avatars of participants themselves in four different contextual backgrounds.	Non-immersive (PC)/interactive	Female undergraduate students with body image concerns (18+)	9/9 (control group not relevant for selected variable)	Self-compassion (SCS-SF)	Purpose of control group unclear. Repeated-measures ANOVA (most likely for experimental group data) shows significant improvements in self-compassion between baseline and post-VR (*p* = 0.000) and between pre-VR and post-VR (*p* = 0.041). No inferential statistics or effect size reported.
Price & Anderson, 2011 [54]	Randomized, longitudinal (between-groups) study	8× [VR exposure]/not reported	No	Challenging public speaking situations, such as classroom or auditorium.	Immersive (HMD)/interactive	Individuals diagnosed with social anxiety disorder (18+)	32/33/25	Ruminations (RQ)	VR exposure (*β* = −8.82, *p* < 0.01) and group CBT (*β* = −9.85, *p* < 0.01) both significantly better than waiting list controls. VR and CBT together had a large effect size compared with waiting list (33% of the variance at post-intervention). After controlling for pre-intervention scores, no significant difference between group CBT and VR at post-intervention (*β* = 0.62, *p* = 0.62).
Riva et al., 2001 [11]	Randomized, longitudinal (between-groups) study	7× once weekly [VR exposure (50)] + low-calorie diet + physical training (minimum twice 30 min walk a week)/350	No	Exposure to environments, potentially eliciting abnormal eating behaviors.	Immersive (HMD)/interactive	Female patients with overweight issues (18+)	28 participants in total (estimated as 14/14)	Assertiveness (AI)	Significant improvements in ability to engage in anxiety-provoking behaviors (*p* = 0.014) within intervention group, and significant improvements in ability to engage in anxiety-provoking behaviors in intervention group compared with control group (*p* = 0.000). No other inferential statistics or effect size reported.
Riva et al., 2002 [55]	Randomized, longitudinal (between-groups) study	7× once weekly [VR exposure (50)] + low-calorie diet + physical training (minimum twice 30 min walk a week)/350	No	Exposure to environments potentially eliciting abnormal eating behaviors.	Immersive (HMD)/interactive	Female patients with binge eating disorder (18+)	20 participants in total (estimated as 10/10)	Assertiveness (AI)	Significant improvements in ability to engage in anxiety-provoking behaviors (*p* = 0.038) within intervention group and non-significant improvements in ability to engage in anxiety-provoking behaviors in intervention group compared with control group (*p* = 0.063). No other inferential statistics and effect size reported.
Roy et al., 2003 [56]	Non-randomized, longitudinal (between-groups) study	Introduction + 11× [VR assessment and/or VR exposure (20)]/220	No	Four different social situations causing anxiety.	Non-immersive (PC)/interactive	Social phobia (18+)	4/6	Assertiveness (RAS)	Based on descriptive statistics, assertiveness increased in both VR and group CBT conditions. No inferential statistics, significance levels, or effect size reported.
Rus-Calafell et al., 2012 [57]	Case study	16× [Content introduction (30) + VR exposure (30)]/480	N/A	Exposure to common daily situations, such as going to a shop or dealing with an angry security guard.	Non-immersive (PC + 3D glasses + headphones)/interactive	Schizophrenia outpatient (18+)	1/-	Assertiveness (AI, number of assertive behaviors observed)	Improvements in ability to engage in anxiety-provoking behaviors pre- and post-intervention (no inferential statistics reported). Significant increase in assertive behaviors pre- to post-intervention (*Z* = −3.28, *p* < 0.05). No effect size reported.
Rus-Calafell et al., 2014 [15]	Longitudinal (within-group) study with 4-month follow-up	16× [Content introduction (30) + VR exposure (30)]/480	N/A	Exposure to common daily situations, such as going to a shop or dealing with an angry security guard.	Non- immersive (PC + 3D glasses + headphones)/interactive	Schizophrenia/schizoaffective disorder outpatients (18+)	12/-	Assertiveness (AI, number of assertive behaviors observed)	Significant improvements in ability to engage in anxiety-provoking behaviors pre- and post- a 4-month follow-up, with large effect [*F*(2, 22) = 70.79, *p* < 0.01, *d* = 0.87]. Same results observed for number of assertive behaviors observed [*F* (3, 33) = 139.76, *p* < 0.01]; no effect size reported.

AI: Assertion Inventory [58]; CMT: Compassionate Mind Training [59]); HMD: Head-Mounted Display; MSCS: Mindfulness Self-Care Scale [43]; PERS: Post-Event Rumination Scale [60,61,62]; RAS: Rathus Assertiveness Schedule [63]; RQ: Rumination Questionnaire [64]; SCIA: Social Contexts Inducing Anxieties [65]; SCCS: Self-compassion and Self-criticism Scales [66]; SCS: Self-Compassion Scale [67]; SCS-SF: Self-compassion Scale Short Form [68]; SOFI: Self-Other Four Immeasurable Scale [44]; T1,T2,T3,T4: Time data collection points; VAS-SC: Visual Analogue Scales for State Changes [69]; VR: Virtual Reality.

**Table 3:** Risk of bias summary for the selected studies.

	Random Sequence Generation/Allocation Concealment(Selection Bias)	Blinding of Participants and Personnel (Performance Bias)	Blinding of Outcome Assessment (Detection Bias)	Incomplete Outcome Data Addressed (Attrition Bias)	Selective Reporting (Reporting Bias)	Other Biases
Ascone et al. (2020) [45]	U	H	H	L	U	H
Cebolla et al., 2019 [42] (7A) (T2,T3)	U	H	H	L	U	H
Cebolla et al., 2019 [42] (7B) (T1,T4)	U	H	H	L	U	H
Falconer et al., 2014 [35]	U	H	H	U	U	H
Falconer et al., 2016 [8]	H	H	H	L	U	H
Hur et al., 2021 [47]	H	H	H	H	U	H
Kim et al., 2020 [48]	H	H	H	H	U	H
Klinger et al., 2004; 2005 [41,40]	H	H	H	L	U	H
Modrego-Alarcón et al., 2021 [49]	L	H	H	U	L	H
Navarrete et al., 2021 [50] (T2,T3)	U	H	H	U	U	H
O’Gara et al., 2022 [51]	H	H	H	H	U	H
Park & Ogle, 2021 [53]	U	H	H	L	U	H
Park et al., 2011 [52]	U	H	H	H	U	H
Price & Anderson, 2011 [54]	U	H	H	H	U	H
Riva et al., 2001 [11]	U	H	H	L	U	H
Riva et al., 2002 [55]	U	H	H	L	U	H
Roy et al., 2003 [56]	H	H	H	L	U	H
Rus-Calafell et al., 2012 [57]	H	H	L	N/A	U	H
Rus-Calafell et al., 2014 [15]	H	H	L	H	U	H

Selection bias was coded as unclear (U) if both random sequence generation and concealment were coded as having unclear risk or if one was coded as unclear and the other as low risk (L). Other bias was coded as high (H) if at least one other bias was identified as high risk (see Appendix B for details).

**Table 4:** GRADE certainty assessment for measured outcomes.

Outcome	Baseline Assessment	Risk of Bias	Inconsistency	Indirectness	Imprecision	Publication Bias	Overall Assessment
Self-criticism/Ruminations	LOW—no randomized controlled trials for this outcome	Downgrading by one point—out of six identified bias categories, all studies show between three and five high risks of bias and one to three unclear risks of bias	No downgrading out of six—five studies report significant results, one study reports non-significant results; effect in all studies points in the same direction	Downgrading by one point—some variability in content and duration of intervention; three out of six studies had no control group for this variable	No downgrading—total number of participants is 232 (<400); however, five studies out of six report large effect size, and one study	No downgrading—results come predominantly from smaller studies; however, this is an emerging area of research, with most studies published since 2016; publication bias possible but not strongly suspected	VERY LOW
Self-compassion/Positive qualities towards self	LOW—only one study out of nine was a randomized controlled trial	Downgrading by one point—out of six identified bias categories, all studies show between three and five high risks of bias and one to three unclear risks of bias	No downgrading—out of nine studies, five report significant results, four report non-significant results; effect in seven studies points in the same direction, one non-significant study points in opposite direction and another non-significant study does not provide information on direction	Downgrading by one point—variability in content, number of interventions, length of exposure, and employed technology; in two out of nine studies, VR did not constitute central part of the intervention	Downgrading by one point—total number of participants is 368 (<400); three studies out of nine did not report effect size, thereby providing insufficient information about effect sizes reported across the studies	No downgrading—results come predominantly from smaller studies; however, this is an emerging area of research, with most studies published since 2016; publication bias possible but not strongly suspected	VERY LOW
Self-protection/Assertiveness	LOW—only one study out of seven was a randomized controlled trial	Downgrading by one point—out of six identified bias categories, all studies show between three and four high risks of bias and one to two unclear risks of bias	No downgrading—out of seven, five studies show significant results, one study does not report significance fully, and one study does not report significance at all; effect in all studies points in the same direction	Downgrading by one point—variability in content, number of interventions, length of exposure and employed technology	Downgrading by one point—total number of participants is 170 (<400); five out of seven studies did not report any effect size, thereby providing insufficient information about effect sizes reported across the studies	Downgrading by one point—results come mainly from smaller studies; none of the studies had been preregistered; only one study reports sample size power calculation; no studies published since 2014; publication bias suspected	VERY LOW

## Appendix A

**Table A1:** Search syntax for selected databases.

Database	Search Topic	Syntax
PubMed	Virtual reality + Self-criticism	(“virtual reality” [Title/Abstract] OR “VR” [Title/Abstract] OR “virtual environment*” [Title/Abstract] OR “virtual world*” [Title/Abstract] OR “avatar” [Title/Abstract] OR “serious game*” [Title/Abstract]) AND (“self-critic*” [Title/Abstract] OR “inner critic*” [Title/Abstract] OR “self-contempt*” [Title/Abstract] OR “self-hat*” [Title/Abstract] OR “self-attack*” [Title/Abstract] OR “ruminat*” [Title/Abstract] OR “self-judg*” [Title/Abstract]) AND (english [Filter])
	Virtual reality + Self-compassion	(“virtual reality” [Title/Abstract] OR “VR” [Title/Abstract] OR “virtual environment*” [Title/Abstract] OR “virtual world*” [Title/Abstract] OR “avatar” [Title/Abstract] OR “serious game*” [Title/Abstract]) AND (“self-compassion*” [Title/Abstract] OR “compassion*” [Title/Abstract] OR “self-sooth*” [Title/Abstract] OR “self-reassur*” [Title/Abstract]) AND (english [Filter])
	Virtual reality + Self-protection	(“virtual reality” [Title/Abstract] OR “VR” [Title/Abstract] OR “virtual environment*” [Title/Abstract] OR “virtual world*” [Title/Abstract] OR “avatar” [Title/Abstract] OR “serious game*” [Title/Abstract]) AND (“self-protecti*” [Title/Abstract] OR “protective anger” [Title/Abstract] OR “assertive anger” [Title/Abstract] OR “assertive*” [Title/Abstract] OR “self-assert*” [Title/Abstract] OR “fierce self-compassion” [Title/Abstract]) AND (english [Filter])
Scopus	Virtual reality + Self-criticism	TITLE-ABS-KEY(“virtual reality” OR “VR” OR “virtual environment*” OR “virtual world*” OR “avatar” OR “serious game*”) AND TITLE-ABS-KEY(“self-critic*” OR “inner critic*” OR “self-contempt*” OR “self-hat*” OR “self-attack*” OR “ruminat*” OR “self-judg*”) AND (LIMIT-TO (LANGUAGE,”English”))
	Virtual reality + Self-compassion	TITLE-ABS-KEY(“virtual reality” OR “VR” OR “virtual environment*” OR “virtual world*” OR “avatar” OR “serious game*”) AND TITLE-ABS-KEY(“self-compassion*” OR “compassion*” OR “self-sooth*” OR “self-reassur*”) AND (LIMIT-TO (LANGUAGE,”English”))
	Virtual reality + Self-protection	TITLE-ABS-KEY(“virtual reality” OR “VR” OR “virtual environment*” OR “virtual world*” OR “avatar” OR “serious game*”) AND TITLE-ABS-KEY(“self-protecti*” OR “protective anger” OR “assertive anger” OR “assertive*” OR “self-assert*” OR “fierce self-compassion”) AND (LIMIT-TO (LANGUAGE,”English”))
Web of Science	Virtual reality + Self-criticism	((TS = (“virtual reality” OR “VR” OR “virtual environment*” OR “virtual world*” OR “avatar” OR “serious game*”)) AND (TS = (“self-critic*” OR “inner critic*” OR “self-contempt*” OR “self-hat*” OR “self-attack*” OR “ruminat*” OR “self-judg*”))) AND LA = (English)
	Virtual reality + Self-compassion	((TS = (“virtual reality” OR “VR” OR “virtual environment*” OR “virtual world*” OR “avatar” OR “serious game*”)) AND (TS = (“self-compassion*” OR “compassion*” OR “self-sooth*” OR “ self-reassur *”))) AND LA = (English)
	Virtual reality + Self-protection	((TS = (“virtual reality” OR “VR” OR “virtual environment*” OR “virtual world*” OR “avatar” OR “serious game*”)) AND (TS = (“self-protecti*” OR “protective anger” OR “assertive anger” OR “assertive*” OR “self-assert*” OR “fierce self-compassion”))) AND LA = (English)

## Appendix B

**Table A2:** Risk of bias analysis for the selected studies.

Reference	Bias	Judgement	Comment
Ascone et al., 2020 [45]	Random sequence generation (selection bias)	Unclear risk	Quote: “…participants were randomized…” Comment: Randomization method unclear.
	Allocation concealment (selection bias)	Unclear risk	Quote: “…participants were randomized…” Comment: Randomization sequence concealment unclear.
	Blinding of participants and personnel (performance bias)	High risk	Quote: “… after participants were randomized and received a psycho-educative video, depending upon their assignment…” Comment: Researchers knew who was in intervention and in control group.
	Blinding of outcome assessment (detection bias)	High risk	Quote: “We used a brief experimental version of the original Self-Compassion Scale…” Comment: Outcome assessment could not be blinded due to self-report measures.
	Incomplete outcome data addressed (attrition bias)	Low risk	Comment: None of the participants dropped out during the study.
	Selective reporting (reporting bias)	Unclear risk	Comment: No protocol available.
	Other Bias: (social desirability bias)	Unclear risk	Comment: Not reported.
	Other Bias: (Hawthorne effect)	Unclear risk	Comment: Unclear whether participants were observed during the intervention.
	Other Bias: (small sample size)	Unclear risk	Quote: “…clear limitation of our study is the small sample size…” Comment: Small sample increases the impact of chance on the results. No power analysis mentioned.
	Other Bias: (other)	High risk	Quote: “ Representativeness of the sample is also questionable, as it mainly consisted of highly educated, young individuals (students).” Comment: The results may be applicable to one specific population only.
	Other Bias: (other)	High risk	Quote: “…we used non-parametric within-group difference analyses…” Comment: Two within-group statistical analyses decrease ecological validity compared with one between-group analysis.
Cebolla et al., 2019 [42]	Random sequence generation (selection bias)	Low risk	Quote: “Participants who fulfilled the inclusion and exclusion criteria were randomly assigned to one of the two study condition…using the Random Allocation Software 2.0.” Comment: Randomization method specified.
	Allocation concealment (selection bias)	Unclear risk	Quote: “Participants who fulfilled the inclusion and exclusion criteria were randomly assigned to one of the two study condition…using the Random Allocation Software 2.0.” Comment: Randomization sequence concealment unclear.
	Blinding of participants and personnel (performance bias)	High risk	Quote: “…to induce this experience, the TMTBA-VR has the support of a performer, a person who is trained to mimic the user’s movements to induce the embodied illusion.” Comment: The intervention was delivered with the help of staff who were part of the intervention.
	Blinding of outcome assessment (detection bias)	High risk	Quote: “Self-Other Four Immeasurable Scale… is a 16-item scale, rated on a five-point Likert scale,” (and) “Mindfulness Self-Care… measures the self-reported frequency of self-care behaviors.” Comment: Outcome assessment could not be blinded due to self-report measures.
	Incomplete outcome data addressed (attrition bias)	Low risk	Comment: None of the participants dropped out during the study.
	Selective reporting (reporting bias)	Unclear risk	Comment: No protocol available.
	Other Bias: (social desirability bias)	Unclear risk	Comment: Not reported.
	Other Bias: (Hawthorne effect)	High risk	Quote: “…to induce this experience, the TMTBA-VR has the support of a performer, a person who is trained to mimic the user’s movements to induce the embodied illusion.” Comment: Participants were aware they were being observed.
	Other Bias: (small sample size)	Unclear risk	Quote: “The size of the sample has been determined with the G-Power program…” Comment: Sample size determined by power calculations. However, this sample size appears to be very small regardless.
	Other Bias: (other)	High risk	Quote: “The sample was composed of 16 students from the University of Valencia…” Comment: The results may be applicable to one specific population only.
	Other Bias: (other)–MSCS	Unclear risk	Quote: “…results…should be viewed with caution because the internal consistencies of these subscales were not adequate.” Comment: Some statistical analysis may be invalid for MSCS measures due to low internal consistencies of two subscales.
Falconer et al., 2014 [35]	Random sequence generation (selection bias)	Unclear risk	Comment: The study followed an experimental design; however, it is not explicitly said that the participants were randomly assigned to given conditions.
	Allocation concealment (selection bias)	Unclear risk	Comment: The study followed an experimental design; however, it is not explicitly said that the participants were randomly assigned to given conditions.
	Blinding of participants and personnel (performance bias)	High risk	Quote: “…participants were guided through the same exercises as before to become accustomed to their environment from this new perspective and, in the 1PP, to their new body. Participants in the 3PP condition did not have a virtual body.” Comment: Researchers were aware of the intervention they were delivering.
	Blinding of outcome assessment (detection bias)	High risk	Quote: “Participants are instructed to imagine, as vividly as possible, that these scenarios are happening to them at the current moment in time and rate on 7-point Likert scales…” Comment: Outcome assessment could not be blinded due to self-report measures.
	Incomplete outcome data addressed (attrition bias)	Unclear risk	Quote: “Three participants were excluded in the 3PP version for technical reasons…” Comment: Three participants out of 24 were excluded from the data analysis due to technical reasons. Attrition happened in one group only, which may have had an impact on the results.
	Selective reporting (reporting bias)	Unclear risk	Comment: No protocol available.
	Other Bias: (social desirability bias)	Unclear risk	Comment: Not reported.
	Other Bias: (Hawthorne effect)	High risk	Quote: “After this, participants were fitted with the head mounted display (HMD) and body tracking suit…” (and) “For the second stage of the experiment participants were seated on one of the stools opposite a seated crying child avatar…” Comment: Participants were likely aware of being monitored during the intervention.
	Other Bias: (other)	High risk	Quote: “Twenty-two females, completed the first person perspective condition and twenty-one females completed the third person perspective condition.” Comment: The results may be applicable to one specific population only.
Falconer et al., 2016 [8]	Random sequence generation (selection bias)	High risk	Comment: No randomization.
	Allocation concealment (selection bias)	High risk	Comment: No randomization.
	Blinding of participants and personnel (performance bias)	High risk	Comment: No control group.
	Blinding of outcome assessment (detection bias)	High risk	Quote: “Participants are instructed to imagine that these scenarios are happening to them now and rate on 7-point scales from…” Comment: Outcome assessment could not be blinded due to self-report measures.
	Incomplete outcome data addressed (attrition bias)	Low risk	Quote: “…1 did not complete due to time commitments and 1 did not complete because she found hearing her voice played back to her aversive.” (and) “We were unable to obtain end-of intervention data from two participants. We therefore repeated the analyses on the entire sample, testing for changes between baseline and follow-up only. These yielded identical findings and are not reported further.” Comment: Missing data addressed.
	Selective reporting (reporting bias)	Unclear risk	Comment: No protocol available.
	Other Bias: (social desirability bias)	Unclear risk	Comment: Not reported.
	Other Bias: (maturation bias)	High risk	Quote: “… the absence of the kind of control condition…” Comment: Some effect of time is possible, as there was approximately a 6-week gap between the first intervention and follow-up, and there was no control group to compare outcomes with.
	Other Bias: (Hawthorne effect)	High risk	Quote: “…participants were asked to close their eyes to complete the body ownership questions, which were recited to them and their responses were recorded by the researcher.” Comment: Participants were likely aware of being monitored during the intervention.
	Other Bias: (small sample size)	Unclear risk	Quote: “…within the context of the study limitations. Chief among these was the relatively small number of patients…” Comment: No power analysis mentioned in the article.
	Other Bias: (chance bias)	High risk	Comment: Potentially small sample, and no control group to compare baseline measures with.
	Other Bias: (other)	High risk	Quote: “Ten patients were currently on antidepressant medication, seven were currently receiving psychological therapy…” Comment: Possible impact of other interventions outside of this study.
	Other Bias: (other)	High risk	Quote: “…the repetition of a single immersive virtual reality scenario…” Comment: The same scenario was repeated three times, which may have led to repetition or boredom.
	Other Bias: (other)	High risk	Quote: “Other limitations include the repeated use of the SCCS, which may have diminished the validity of the measure…” Comment: The same questionnaire was used three times, which may have led to its diminished validity.
	Other Bias: (other)	High risk	Quote: “…the absence of the kind of control condition we previously employed…” Comment: Lack of control group may lead to overestimation of results.
Hur et al., 2021 [47]	Random sequence generation (selection bias)	High risk	Quote: “We recruited individuals with and without social anxiety disorder…” Comment: No randomization.
	Allocation concealment (selection bias)	High risk	Quote: “We recruited individuals with and without social anxiety disorder…” Comment: No randomization.
	Blinding of participants and personnel (performance bias)	High risk	Quote: “The researchers stayed with the participants throughout the VR sessions to address emergencies such as extreme anxiety or panic attacks.” Comment: Researchers knew who was in intervention group.
	Blinding of outcome assessment (detection bias)	High risk	Quote: “All participants…completing self-report questionnaires, including… Post-Event Rumination Scale…” Comment: Outcome assessment could not be blinded due to self-report measures.
	Incomplete outcome data addressed (attrition bias)	High risk	Quote: “We initially recruited and enrolled 40 individuals with social anxiety disorder… 21 participants completed the VR session and postintervention assessments.” Comment: High attrition rate.
	Selective reporting (reporting bias)	Unclear risk	Comment: Outcome measures not fully defined in the protocol.
	Other Bias: (social desirability bias)	Unclear risk	Comment: Not reported.
	Other Bias: (Hawthorne effect)	High risk	Quote: “The researchers stayed with the participants throughout the VR sessions to address emergencies such as extreme anxiety or panic attacks.” Comment: Participants were likely aware they were being observed.
	Other Bias: (small sample size)	Unclear risk	Quote: “The recommended sample size for a task fMRI is 20; our sample size (n = 25 in the social anxiety disorder group at baseline, n = 21 in the social anxiety disorder group at follow-up) seemed to have adequate statistical power.” Comment: No power calculation for sample size, only a recommendation.
	Other Bias: (other)	High risk	Quote: “From the second session onward, the participants were asked to select the level of difficulty they desired.” Comment: Intervention appears, to some extent, inconsistent across the intervention group.
	Other Bias: (other)	High risk	Quote: “…the case-control study using sham was not properly performed. Therefore, there is a limitation in interpretation to determine the effect of VR therapy through this study. Second, the lack of a direct comparison with conventional face-to-face therapy should be considered when interpreting these findings.” Comment: Lack of control group for rumination scores may lead to overestimation of results.
Kim et al., 2020 [48]	Random sequence generation (selection bias)	High risk	Quote: “We recruited individuals for the SAD and healthy control groups…” Comment: No randomization.
	Allocation concealment (selection bias)	High risk	Quote: “We recruited individuals for the SAD and healthy control groups…” Comment: No randomization.
	Blinding of participants and personnel (performance bias)	High risk	Quote: “Researchers were present throughout the VR experience to deal with any unexpected situations.” Comment: Researchers knew who was in intervention group.
	Blinding of outcome assessment (detection bias)	High risk	Quote: “…this study employed self-rated scales that could be confounded by bias…” Comment: Outcome assessment could not be blinded due to self-report measures.
	Incomplete outcome data addressed (attrition bias)	High risk	Quote: “…8 patients with SAD and 1 healthy control participant dropped out for personal reasons (e.g., time constraints).” Comment: Potentially high and uneven attrition rate [8 out of 40 (20%) in intervention group].
	Selective reporting (reporting bias)	Unclear risk	Comment: Outcome measures not fully defined in the protocol.
	Other Bias: (social desirability bias)	Unclear risk	Comment: Not reported.
	Other Bias: (Hawthorne effect)	High risk	Quote: “Researchers were present throughout the VR experience to deal with any unexpected situations.” Comment: Participants were likely aware they were being observed.
	Other Bias: (small sample size)	Unclear risk	Quote: “…a total of 32 patients with SAD and 33 healthy control participants completed this study.” Comment: No power calculation mentioned in the article.
	Other Bias: (other)	High risk	Quote: “During the second session, participants could select and proceed to their desired level.” Comment: Intervention appears, to some extent, inconsistent across the intervention group.
	Other Bias: (other)	High risk	Quote: “Participants answered the self-reported psychological scales four times: at baseline (before the VR experience), after the second VR session, after the fourth session, and after termination (i.e., after the sixth session).” Comment: The same questionnaire was used four times, which may have led to its diminished validity.
	Other Bias: (other)	High risk	Quote: “…this study did not have a sham or waitlist control group, which limits interpretation of the results.” Comment: Lack of control group may lead to overestimation of results.
Klinger et al., 2004; 2005 [41,40]	Random sequence generation (selection bias)	High risk	Quote: “Participants in the two conditions were matched based on the following variables: gender, age, duration, severity of social phobia…, ability to use computers or virtual reality software, and time availability for some groups that were already prescheduled.” Comment: No randomization.
	Allocation concealment (selection bias)	High risk	Quote: “Participants in the two conditions were matched based on the following variables: gender, age, duration, severity of social phobia…, ability to use computers or virtual reality software, and time availability for some groups that were already prescheduled.” Comment: No randomization.
	Blinding of participants and personnel (performance bias)	High risk	Quote: “Each session was individual and directed by a cognitive behavior therapist.” Comment: The therapists were present during the intervention and likely knew they were delivering an actual intervention.
	Blinding of outcome assessment (detection bias)	High risk	Quote: “The Rathus Assertiveness Schedule is a self-report that measures social assertiveness.” Comment: Outcome assessment could not be blinded due to self-report measures.
	Incomplete outcome data addressed (attrition bias)	Low risk	Comment: None of the participants dropped out during the study.
	Selective reporting (reporting bias)	Unclear risk	Comment: No protocol available.
	Other Bias: (social desirability bias)	Unclear risk	Comment: Not reported.
	Other Bias: (Hawthorne effect)	High risk	Quote: “Each session was individual and directed by a cognitive behavior therapist.” Comment: The therapist was present during the study, so participants were aware they were being observed.
	Other Bias: (chance bias)	Unclear risk	Quote: “Participants in the two conditions were matched based on the following variables: gender, age, duration, severity of social phobia…, ability to use computers or virtual reality software, and time availability for some groups that were already prescheduled.” Comment: Participants appear to be manually matched rather than randomly split.
Modrego-Alarcón et al., 2021 [49]	Random sequence generation (selection bias)	Low risk	Quote: “Assignation of the subjects was performed… through a simple randomization sequence generated by computer.” Comment: Randomization method specified.
	Allocation concealment (selection bias)	Low risk	Quote: “Assignation of the subjects was performed after the baseline evaluation by a member of the research group, who had no knowledge about the study aims and was not involved in the study in any other way…” Comment: Allocation process was blinded.
	Blinding of participants and personnel (performance bias)	High risk	Quote: “…providers and participants were able to know what kind of intervention they were offering and receiving, respectively…” Comment: Researchers knew what intervention they were delivering.
	Blinding of outcome assessment (detection bias)	High risk	Quote: “…the assessment was conducted using self-reported measures, which could introduce some bias…” Comment: Outcome assessment could not be blinded due to self-report measures.
	Incomplete outcome data addressed (attrition bias)	Unclear risk	Quote: “‘MBP + VR’ had a significantly higher number of participants who attended 3 or more sessions: 89 (95.7%), compared to 77 (82.8%) in the ‘MBP’ group, and 66 (70.2%) in the ‘Relaxation’ group (Fisher *p* < .001).” Comment: Overall, 92 of 280 (33%) participants dropped out between baseline and follow-up. There is a big difference in drop-out rates across the three conditions. It was, however, part of the intervention to increase adherence, so rather than an uneven drop-out rate, this could be the effect of the intervention.
	Selective reporting (reporting bias)	Low risk	Comment: A number of secondary measures defined in the registered protocol were not reported in the published study; this is not the case for self-compassion, though, which is one of the domains of this systematic review.
	Other Bias: (social desirability bias)	Unclear risk	Comment: Not reported.
	Other Bias: (Hawthorne effect)	High risk	Quote: “…the implementation of VR exercises was performed by another clinical psychologist…” Comment: Participants were likely aware they were being observed.
	Other Bias: (other)	High risk	Quote: “…the sample was mostly comprised of females, students of health-related degrees and from only one city (Zaragoza, Spain), which implies that our results should not be considered representative of the whole Spanish undergraduate population.” Comment: The results may be applicable to one specific population only.
Navarrete et al., 2021 [50]	Random sequence generation (selection bias)	Low risk	Quote: “Participants who met the inclusion and exclusion criteria were randomly assigned to one of the two study conditions…using Random Allocation Software 2.0.” Comment: Randomization method specified.
	Allocation concealment (selection bias)	Unclear risk	Quote: “Participants who met the inclusion and exclusion criteria were randomly assigned to one of the two study conditions…using Random Allocation Software 2.0.” Comment: Randomization sequences concealment not specified.
	Blinding of participants and personnel (performance bias)	High risk	Quote: “Two researchers were present in all sessions to run the experiment…” Comment: The researchers were present during the intervention and knew they were delivering an actual intervention.
	Blinding of outcome assessment (detection bias)	High risk	Quote: “… state self-compassion…levels were assessed with the Visual analogue scales…” Comment: Outcome assessment could not be blinded due to self-report measures.
	Incomplete outcome data addressed (attrition bias)	Unclear risk	Comment: Three participants out of 44 dropped out—one out of 22 in the intervention group and two out of 22 in the control group. The drop-out rate could be considered even, but overall it is more than 5%.
	Selective reporting (reporting bias)	Unclear risk	Comment: Protocol not available.
	Other Bias: (social desirability bias)	Unclear risk	Comment: Not reported.
	Other Bias: (Hawthorne effect)	High risk	Quote: “Two researchers were present in all sessions to run the experiment…” Comment: Participants were likely aware they were being observed.
	Other Bias: (other)	High risk	Quote: “Professionals from healthcare institutions or healthcare students were invited to participate in a study aimed at increasing their compassionate skills toward their patients.” Comment: The results may be applicable to one specific population only.
O’Gara et al., 2022 [51]	Random sequence generation (selection bias)	High risk	Quote: “Potential participants were identified by clinical teams, and a diverse convenience sample undergoing a range of cancer treatments across tumour types from one specialist centre recruited.” Comment: No randomization.
	Allocation concealment (selection bias)	High risk	Quote: “Potential participants were identified by clinical teams, and a diverse convenience sample undergoing a range of cancer treatments across tumour types from one specialist centre recruited.” Comment: No randomization.
	Blinding of participants and personnel (performance bias)	High risk	Comment: No control group.
	Blinding of outcome assessment (detection bias)	High risk	Quote: “The SCS is a 26-item instrument that measures self-compassion… according to a five-point scale (1 = almost never; 5 = almost always).” Comment: Outcome assessment could not be blinded due to self-report measures.
	Incomplete outcome data addressed (attrition bias)	High risk	Quote: “Acceptability of the intervention was deemed satisfactory as >60% (N = 13; 65%) of participants completed all three sessions.” Comment: However, only 10 participants completed SCS according to article’s supplementary tables—the reasons for further dropouts are unclear.
	Selective reporting (reporting bias)	Unclear risk	Comment: Protocol not available.
	Other Bias: (social desirability bias)	Unclear risk	Comment: Not reported.
	Other Bias: (maturation bias)	High risk	Quote: “… planned appointments, spaced at least a week apart.” Comment: The average duration of full VR treatment unclear. Additionally, no control group for comparison.
	Other Bias: (Hawthorne effect)	Unclear risk	Comment: Unclear whether researchers were present while patients underwent the intervention.
	Other Bias: (small sample size)	Unclear risk	Quote: “The small sample did not allow for adjustment of confounding variables in the quantitative analysis so that any notable differences in baseline characteristics or response to the intervention in the study population could be identified.” Comment: Small sample. Additionally, no power calculation reported.
	Other Bias: (chance bias)	High risk	Comment: Potentially small sample and no control group to compare collected scores with.
	Other Bias: (other)	High risk	Comment: The same questionnaire was used four times, which may have led to its diminished validity.
Park & Ogle, 2021 [53]	Random sequence generation (selection bias)	Unclear risk	Quote: “Each participant was randomly assigned to one of the study groups…” Comment: Randomization method not specified.
	Allocation concealment (selection bias)	Unclear risk	Quote: “Each participant was randomly assigned to one of the study groups…” Comment: Randomization sequence concealment not specified.
	Blinding of participants and personnel (performance bias)	High risk	Quote: “When each participant entered the room, a researcher offered her a bottle of water, demonstrated the avatar viewing tools for her, and allowed her to practice until she felt comfortable.” Comment: Researchers knew they were delivering an intervention.
	Blinding of outcome assessment (detection bias)	High risk	Quote: “…computed subscale scores on a 5-point Likert scale ranging from almost never (1) to almost always (5), to generate a total score of self-compassion.” Comment: Outcome assessment could not be blinded due to self-report measures.
	Incomplete outcome data addressed (attrition bias)	Low risk	Comment: None of the participants dropped out during the study.
	Selective reporting (reporting bias)	Unclear risk	Comment: No protocol available.
	Other Bias: (social desirability bias)	Unclear risk	Comment: Not reported.
	Other Bias: (small sample size)	Unclear risk	Quote: “…we recruited eighteen female adults…” Comment: No power calculation reported.
	Other Bias: (other)	High risk	Quote: “The total duration of completing the virtual avatar experience ranged from 4 to 13 min.” Comment: The VR intervention may have been inconsistent across the participants.
	Other Bias: (other)	High risk	Quote: “…we recruited a convenient sample of young college female students with some level of body image concern from a U.S. 4-year university, representing a single geographical location.” Comment: The results may be applicable to one specific population only.
	Other Bias: (other)	High risk	Comment: The study appears not to have included the control group in the analysis.
Park et al., 2011 [52]	Random sequence generation (selection bias)	Unclear risk	Quote: “…they were randomly assigned to either SST-TR (n = 45) or SST-VR (n = 46).” Comment: Randomization method not specified.
	Allocation concealment (selection bias)	Unclear risk	Quote: “…they were randomly assigned to either SST-TR (n = 45) or SST-VR (n = 46).” Comment: Randomization sequence concealment not specified.
	Blinding of participants and personnel (performance bias)	High risk	Quote: “Every session included a therapist modeling followed by the participant’s role-playing…” Comments: Researchers knew they were delivering an intervention.
	Blinding of outcome assessment (detection bias)	High risk	Quote: “For secondary outcomes, we selected three self-reports…” Comment: Outcome assessment could not be blinded due to self-report measures.
	Incomplete outcome data addressed (attrition bias)	High risk	Quote: “…no difference between the two groups in the drop-out rate…” (and) “All missing values in the post-session questionnaire and test were due to the participants’ absences. These were substituted by a within-group average.” Comment: A total of 28 out of 91 (31%) participants dropped out.
	Selective reporting (reporting bias)	Unclear risk	Comment: No protocol available.
	Other Bias: (social desirability bias)	Unclear risk	Comment: Not reported.
	Other Bias: (Hawthorne effect)	High risk	Quote: “Every session included a therapist modeling followed by the participant’s role-playing…” Comments: Participants were aware they were observed.
	Other Bias: (small sample size)	Unclear risk	Quote: “This study enrolled 91 participants…” Comment: No power calculation for sample size reported.
	Other Bias: (other)	High risk	Quote: “This study enrolled 91 participants with schizophrenia who were all inpatients of the Severance Mental Health Hospital, Yonsei University College of Medicine.” Comment: The results may be applicable to one specific population only.
Price & Anderson, 2011 [54]	Random sequence generation (selection bias)	Low risk	Quote: “The randomization procedure was administered by the project coordinator, using a random number generator according to ID number.” Comment: Randomization method specified.
	Allocation concealment (selection bias)	Unclear risk	Quote: “Participants were randomly assigned using a simple randomization procedure.” Comment: Randomization sequence concealment not specified.
	Blinding of participants and personnel (performance bias)	High risk	Quote: “The therapist was able to communicate with the participant through a microphone to encourage sustained contact with the feared stimuli.” Comment: The therapist was aware that s/he was delivering an intervention.
	Blinding of outcome assessment (detection bias)	High risk	Quote: “The RQ is a 5-item self-report questionnaire…” Comment: Outcome assessment could not be blinded due to self-report measures.
	Incomplete outcome data addressed (attrition bias)	High risk	Comment: A total of 26 of 91 (29%) participants dropped out during the study; 20% in intervention and 35% in control condition. High and uneven drop-out rate.
	Selective reporting (reporting bias)	Unclear risk	Comment: No protocol available.
	Other Bias: (social desirability bias)	Unclear risk	Comment: Not reported.
	Other Bias: (Hawthorne effect)	High risk	Quote: “The therapist was able to communicate with the participant through a microphone to encourage sustained contact with the feared stimuli.” Comment: Participants were likely aware they were being observed.
	Other Bias: (small sample size)	Unclear risk	Quote: “Participants were 91 individuals diagnosed with social anxiety.” Comment: No power calculation for sample size reported.
	Other Bias: (recall bias)	High risk	Quote: “The current study examined PEP for the previous week at the end of an exposure therapy session, which renders it vulnerable to recall bias.” Comment: Potential interference from current exposure.
	Other Bias: (other)	High risk	Quote: “Participants were given self-report measures prior to being randomized to a condition (pretreatment), at the end of the fourth session (mid-treatment), and at the end of the eighth session (posttreatment).” Comment: The same questionnaire was used three times, which may have led to its diminished validity.
	Other Bias: (sampling bias)	High risk	Quote: “Also, the rate of co-morbidity in the current sample (12%) deserves mention, as it is lower than what is typically found for individuals with social anxiety disorder.” Comment: Results may not be applicable to all individuals with social anxiety disorder.
Riva et al., 2001 [11]	Random sequence generation (selection bias)	Unclear risk	Quote: “The sample was randomly divided into two groups…” Comment: Randomization method not specified.
	Allocation concealment (selection bias)	Unclear risk	Quote: “The sample was randomly divided into two groups…” Comment: Randomization sequence concealment not specified.
	Blinding of participants and personnel (performance bias)	High risk	Quote: “In all the sessions, the therapists follow the Socratic style: they use a series of questions, related to the contents of the virtual environment, to help clients synthesize information and reach conclusions on their own.” Comment: Therapists know they are delivering an intervention.
	Blinding of outcome assessment (detection bias)	High risk	Quote: “Italian version…of the assertion inventory (AI)…” Comment: Outcome assessment could not be blinded due to self-report measures.
	Incomplete outcome data addressed (attrition bias)	Low risk	Comment: None of the participants dropped out during the study.
	Selective reporting (reporting bias)	Unclear risk	Comment: No protocol available.
	Other Bias: (social desirability bias)	Unclear risk	Comment: Not reported.
	Other Bias: (Hawthorne effect)	High risk	Quote: “In all the sessions, the therapists follow the Socratic style: they use a series of questions, related to the contents of the virtual environment, to help clients synthesize information and reach conclusions on their own.” Comment: Participants knew they were being observed.
	Other Bias: (small sample size)	Unclear risk	Quote: “The individuals included were 28 women…” Comment: No power analysis for sample size reported.
Riva et al., 2002 [55]	Random sequence generation (selection bias)	Unclear risk	Quote: “The sample was randomly divided into two groups…” Comment: Randomization method not specified.
	Allocation concealment (selection bias)	Unclear risk	Quote: “The sample was randomly divided into two groups…” Comment: Randomization sequence concealment not specified.
	Blinding of participants and personnel (performance bias)	High risk	Quote: “In all the sessions, the therapists follow the Socratic style: they use a series of questions, related to the contents of the virtual environment, to help clients synthesize information and reach conclusions on their own.” Comment: Therapist know they are delivering an intervention.
	Blinding of outcome assessment (detection bias)	High risk	Quote: “Italian version…of the assertion inventory (AI)…” Comment: Outcome assessment could not be blinded due to self-report measures.
	Incomplete outcome data addressed (attrition bias)	Low risk	Comment: None of the participants dropped out during the study.
	Selective reporting (reporting bias)	Unclear risk	Comment: No protocol available.
	Other Bias: (social desirability bias)	Unclear risk	Comment: Not reported.
	Other Bias: (Hawthorne effect)	High risk	Quote: “In all the sessions, the therapists follow the Socratic style: they use a series of questions, related to the contents of the virtual environment, to help clients synthesize information and reach conclusions on their own.” Comment: Participants knew they were being observed.
	Other Bias: (small sample size)	Unclear risk	Quote: “The individuals included were 20 women…” Comment: No power analysis for sample size reported.
Roy et al., 2003 [56]	Random sequence generation (selection bias)	High risk	Comment: No randomization.
	Allocation concealment (selection bias)	High risk	Comment: No randomization.
	Blinding of participants and personnel (performance bias)	High risk	Quote: “Each session is individual and directed by a cognitive-behavioral psychotherapist.” Comment: The therapist was present during the study, so s/he was aware of delivering an intervention.
	Blinding of outcome assessment (detection bias)	High risk	Quote: “The Rathus Assertiveness Schedule is a self-report questionnaire enabling to measure the degree of assertiveness.” Comment: Outcome assessment could not be blinded due to self-report measures.
	Incomplete outcome data addressed (attrition bias)	Low risk	Comment: None of the participants dropped out during the study.
	Selective reporting (reporting bias)	Unclear risk	Comment: No protocol available.
	Other Bias: (social desirability bias)	Unclear risk	Comment: Not reported.
	Other Bias: (Hawthorne effect)	High risk	Quote: “Each session is individual and directed by a cognitive-behavioral psychotherapist.” Comment: The therapist was present during the study, so participants were aware they were being observed.
	Other Bias: (small sample size)	Unclear risk	Quote: “A preliminary study compared 10 subjects…” Comment: No power calculation reported.
Rus-Calafell et al., 2012 [57]	Random sequence generation (selection bias)	High risk	Comment: A case study with only one participant.
	Allocation concealment (selection bias)	High risk	Comment: A case study with only one participant.
	Blinding of participants and personnel (performance bias)	High risk	Comment: A case study with only one participant.
	Blinding of outcome assessment (detection bias)	Low risk	Quote: “The self-report score for assertiveness (AI) also showed…” (and) “…assertive behaviors (measured by a specific social interaction activity)…” Comment: Apart from self-report, there were also behavioral measures used to assess the intervention.
	Incomplete outcome data addressed (attrition bias)	N/A	Comment: A case study with only one participant.
	Selective reporting (reporting bias)	Unclear risk	Comment: No protocol available.
	Other Bias: (social desirability bias)	Unclear risk	Comment: Not reported.
	Other Bias: (maturation bias)	High risk	Quote: “The treatment consisted of 16 twice-weekly sessions…” Comment: Time may have played some role as the treatment lasted 8 weeks. No control group.
	Other Bias: (Hawthorne effect)	High risk	Quote: “…the therapist and the patient dealt with social anxiety and interpersonal interactions…” Comment: The patient knew she was being observed.
	Other Bias: (small sample size)	High risk	Comment: A case study with only one participant.
	Other Bias: (chance bias)	High risk	Comment: A case study with only one participant.
Rus-Calafell et al., 2014 [15]	Random sequence generation (selection bias)	High risk	Comment: No randomization.
	Allocation concealment (selection bias)	High risk	Comment: No randomization.
	Blinding of participants and personnel (performance bias)	High risk	Comment: No control group. Researchers knew they were delivering an intervention.
	Blinding of outcome assessment (detection bias)	Low risk	Quote: “Assertion inventory (…). This is a self-reported questionnaire…” (and) “Assertive behaviors. This score comprises the number of correct emitted assertive behaviors and the correct identification of assertive, passive and aggressive behaviors/communication styles of others…” Comment: Apart from self-report, also behavioral measures used to assess the intervention.
	Incomplete outcome data addressed (attrition bias)	High risk	Quote: “Fifteen patients were enrolled, and twelve completed the study.” (and) “Three of the participants abandoned the study because they missed more than 3 consecutive sessions; their reported reasons were as follows: illness, schedule incompatibilities and forgetfulness.” Comment: An amount of 20% attrition may have affected the outcomes.
	Selective reporting (reporting bias)	Unclear risk	Comment: No protocol available.
	Other Bias: (social desirability bias)	Unclear risk	Comment: Not reported.
	Other Bias: (maturation bias)	High risk	Quote: “The therapy took place across sixteen “one-on-one” sessions, conducted twice a week over eight weeks.” (and) “After the complementation of the treatment, the post-assessment was performed, and the patients were given an appointment four months later for the follow-up assessment.” Comment: The whole procedure lasted 6 months, so time may have played some part in the outcomes. No control group involved.
	Other Bias: (Hawthorne effect)	High risk	Quote: “At the end of the intervention, participants were asked to complete an anonymous satisfaction questionnaire and rate their perception of (…) the therapist’s work…” Comment: Participants likely knew they were being observed.
	Other Bias: (small sample size)	Unclear risk	Quote: “Fifteen patients were enrolled, and twelve completed the study.” Comment: No power calculation for sample size reported.
	Other Bias: (chance bias)	Unclear risk	Quote: “The reported results are based on a small, uncontrolled pilot study.” Comment: Potentially small sample and no control group to compare baseline measures with.
